# Standardized Computer-Assisted Analysis of 5-hmC Immunoreactivity in Dysplastic Nevi and Superficial Spreading Melanomas

**DOI:** 10.3390/ijms241914711

**Published:** 2023-09-28

**Authors:** Elias A. T. Koch, Carola Berking, Ramona Erber, Michael Erdmann, Franklin Kiesewetter, Stefan Schliep, Markus V. Heppt

**Affiliations:** 1 Department of Dermatology, Uniklinikum Erlangen, Friedrich-Alexander-University Erlangen-Nürnberg (FAU), 91054 Erlangen, Germany; carola.berking@uk-erlangen.de (C.B.); michael.erdmann@uk-erlangen.de (M.E.);; 2 Comprehensive Cancer Center Erlangen—European Metropolitan Area of Nuremberg (CCC ER-EMN), 91054 Erlangen, Germany; ramona.erber@uk-erlangen.de; 3 Bavarian Cancer Research Center (BZKF), 91054 Erlangen, Germany; 4 Institute of Pathology, Uniklinikum Erlangen, Friedrich-Alexander-University Erlangen-Nürnberg (FAU), 91054 Erlangen, Germany; 5 MVZ Pathology, Sozialstiftung Bamberg, 96049 Bamberg, Germany; franklin.kiesewetter@fau.de

**Keywords:** 5-hmC, melanoma, dysplastic nevus, immunohistochemistry, computer-assisted analysis

## Abstract

5-Hydroxymethylcytosine (5-hmC) is an important intermediate of DNA demethylation. Hypomethylation of DNA is frequent in cancer, resulting in deregulation of 5-hmC levels in melanoma. However, the interpretation of the intensity and distribution of 5-hmC immunoreactivity is not very standardized, which makes its interpretation difficult. In this study, 5-hmC-stained histological slides of superficial spreading melanomas (SSM) and dysplastic compound nevi (DN) were digitized and analyzed using the digital pathology and image platform QuPath. Receiver operating characteristic/area under the curve (ROCAUC) and t-tests were performed. A *p*-value of <0.05 was used for statistical significance, and a ROCAUC score of >0.8 was considered a “good” result. In total, 92 5-hmC-stained specimens were analyzed, including 42 SSM (45.7%) and 50 DN (54.3%). The mean of 5-hmC-positive cells/mm^2^ for the epidermis and dermo-epidermal junction and the entire lesion differed significantly between DN and SSM (*p* = 0.002 and *p* = 0.006, respectively) and showed a trend towards higher immunoreactivity in the dermal component (*p* = 0.069). The ROCAUC of 5-hmC-positive cells of the epidermis and dermo-epidermal junction was 0.79, for the dermis 0.74, and for the entire lesion 0.76. These results show that the assessment of the epidermal with junctional expression of 5-hmC is slightly superior to dermal immunoreactivity in distinguishing between DN and SSM.

## 1. Introduction

Melanoma is an aggressive malignant tumor and represents one of the most lethal skin cancers. Surgical excision and histological examination of conspicuous pigmented lesions on dermoscopy is the gold standard. Melanoma arises from the melanocytes of the neural crest, has a variety of genetic, and possibly epigenetic, alterations, and appears in a broad variety of histologic and cytomorphologic features. Many different variants are reported. Furthermore, some melanomas lack melanin, which may complicate correct diagnosis [1]. A major challenge faced by virtually all practicing clinicians and dermatopathologists is the reliable distinction between dysplastic nevi and melanomas. The histological diagnosis is traditionally performed by conventional hematoxylin and eosin (HE) stain via assessment of morphological features of the lesion, including pagetoid spread, cytological atypia, presence of dermal mitoses, asymmetry, lack of circumscription, impaired maturation, and hypercellularity [2]. Nevertheless, in over 50% of cases, immunohistochemical (IHC) markers are used to support the diagnosis [3]. Immunohistochemistry is a straightforward and cost-effective method for the assessment of biomarker expression in routinely processed tumor tissue sections. Despite the development of novel techniques of molecular pathology and the increasingly wide availability of next-generation sequencing, IHC remains the most valuable ancillary tool for ambiguous melanocytic lesions. However, the process of translating an IHC biomarker into clinical practice requires several steps, and numerous studies have focused their attention on molecules, which can be used in differential diagnoses [1,2]. Melan-A (also MART-1) and Sry-related HMg-Box gene 10 (SOX-10) are commonly used to stain all melanocytic cells; both are sensitive and specific markers for melanocytic lesions [2,4]. Further IHC markers have been established to aid in the differential diagnosis. Notably, expression of preferentially expressed antigen in melanoma (PRAME) and p53 (also a helpful marker for desmoplastic melanoma) are significantly higher in melanomas than in nevi [5,6,7]. On the other hand, 5-hydroxymethylcytosine (5-hmC) shows an intense immunoreactivity in benign nevus cells, whereas it is increasingly lost in dysplastic melanocytic nevi (DN) and frequently absent in melanomas [8,9]. Also, p16 shows a decreased nuclear staining within melanomas compared with nevi [10]. Taking advantage of these markers, the distinction between melanoma and nevi is possible in most cases, with no need for other ancillary methods such as molecular pathology [2].

Epigenetic changes play a significant role in the development and progression of melanoma. DNA methylation is among the most well-studied epigenetic alterations that involve the addition or removal of methyl groups, typically at cytosine residues in CpG dinucleotides. Hypermethylation of CpG islands in gene promoter regions, which are usually rich in CpG dinucleotides, inhibits the binding of transcription factors and RNA polymerase to the promoter, thereby inhibiting transcription initiation and resulting in gene silencing. Epigenetic silencing has specifically been reported for important tumor suppressors in melanoma such as CDKN2A (p16) and CDKN2B (p15). Furthermore, changes in DNA methylation can affect the chromatin structure inducing a more condensed chromatin state that is less accessible for RNA polymerases and chromatin transcription. Finally, DNA methylation patterns may even reach a more stable state and be inherited in cell divisions, possibly leading to long-term gene repression.

To this end, 5-hmC is produced through the enzymatic conversion of 5-methylcytosine by the ten-eleven translocation family of DNA hydroxylases (TET enzymes) and is an important intermediate in the DNA demethylation process. DNA methylation at the fifth position of cytosine is an important epigenetic marker, which is crucial for a variety of biological processes. In general, alterations in DNA methylation are frequent in cancer, and 5-hmC levels are reduced in solid tumors compared with normal tissues [11]. The absence of 5-hmC facilitates abnormal expression of oncogenes, resulting in uncontrolled cell proliferation and the acquisition of malignant characteristics [12]. Mechanistically, the loss of 5-hmC may be associated with increased DNA hypermethylation and silencing of critical genes involved in tumor suppression and cell differentiation. Studies indicate that lower levels of 5-hmC are associated with advanced-stage melanomas and are predictive of worse patient outcomes [12,13]. These include reduced survival rates and an increased risk of disease recurrence. Melanoma is a highly aggressive form of skin cancer, exhibiting a significant loss of 5-hmC levels at all stages of melanomas, including primary melanomas, metastases, conjunctival melanomas, mucosal melanomas, and sentinel lymph node biopsies [14]. Merely acral melanoma did not show a significant loss of expression 5-hmC. The diagnostic accuracy increased when PRAME, 5-hmC, and p53 were used in combination [7].

However, the interpretation of the intensity and distribution of 5-hmC immunostaining is not very standardized, which makes its interpretation difficult. Typically, a manual immunostain score of a board-certified dermatopathologist is performed according to the proportion of cells with a positive stain [13]. In the first pioneering reports on 5-hmC ICH in melanocytic lesions, immunoreactivity was scored semiquantitatively based on the nuclear staining intensity from 0 to 3 (0: absent staining; 1 = faint staining; 2 = medium staining; 3 = fuscia). Subsequently, the percentage of 5-hmC-positive cells was counted in five randomly selected high-power fields (1 mm^2^), and the average was calculated. In the case of heterogeneous staining, the predominant staining pattern was numerically assessed. Although this approach is widely followed in daily practice and represents the current gold standard of 5-hmC assessment, this method is not a precise measurement, may contain bias as the investigator can recognize the diagnosis of the samples due to the overall morphology, and includes an intra- and inter-observer variability. Thus, standardized computer-based assessment is highly desirable. In this study, we evaluated a standardized computer-assisted assessment of 5-hmC staining to distinguish between thin superficial spreading melanomas (SSMs) and DN.

## 2. Results

**Figure 1:**
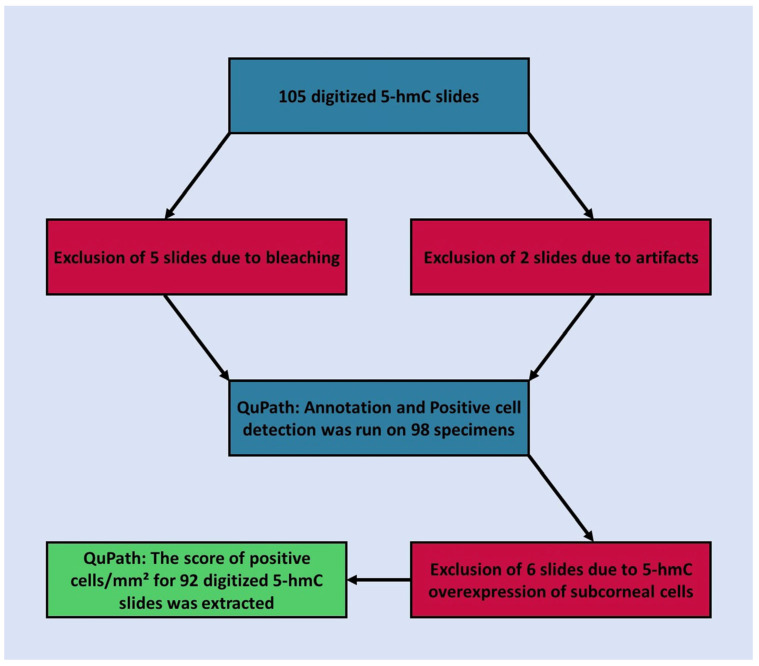
Individual work steps before the extraction, measurements, and statistical analysis of the final analysis. The exclusion of slides was necessary to create a homogeneous group of specimens in terms of immunohistochemical reactivity and high quality.

**Figure 2:**
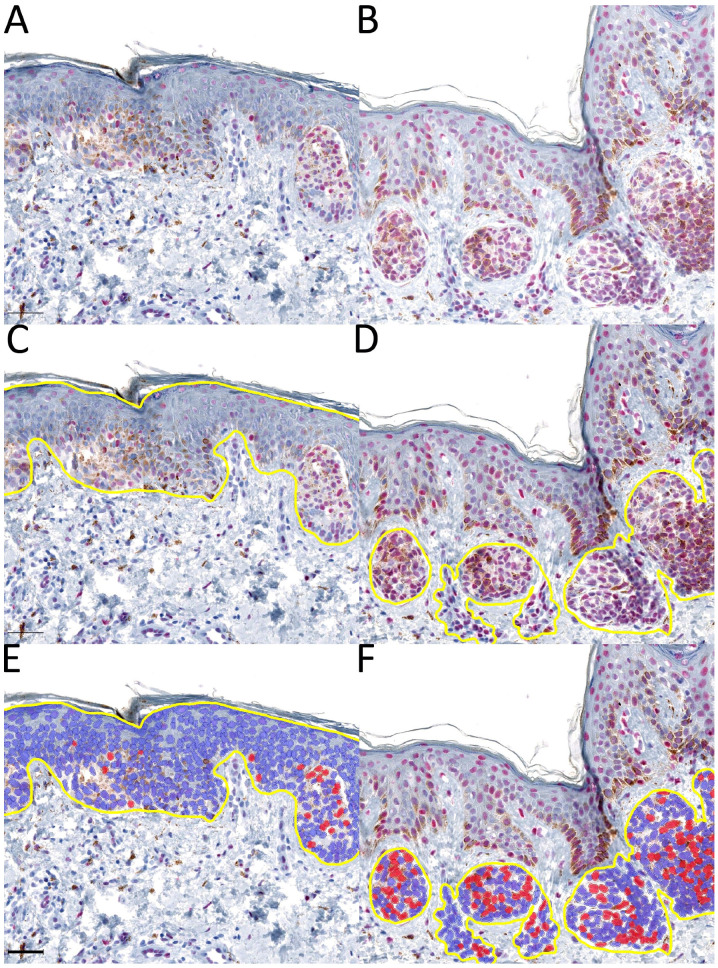
Annotation and positive-cell detection preceding the standardized assessment of 5-hmC with Qupath. Here, (**A**,**B**) represents two dysplastic nevi without annotation, (**C**,**D**) the annotation of the area of interest ((**C**): epidermis and dermo-epidermal junction; (**D**): dermal component), and (**E**,**F**) the positive-cell detection after setting a threshold (the blue cells are considered to be 5-hmC-negative and the red cells to be 5-hmC-positive). Black scale bar = 50 µm.

**Figure 3:**
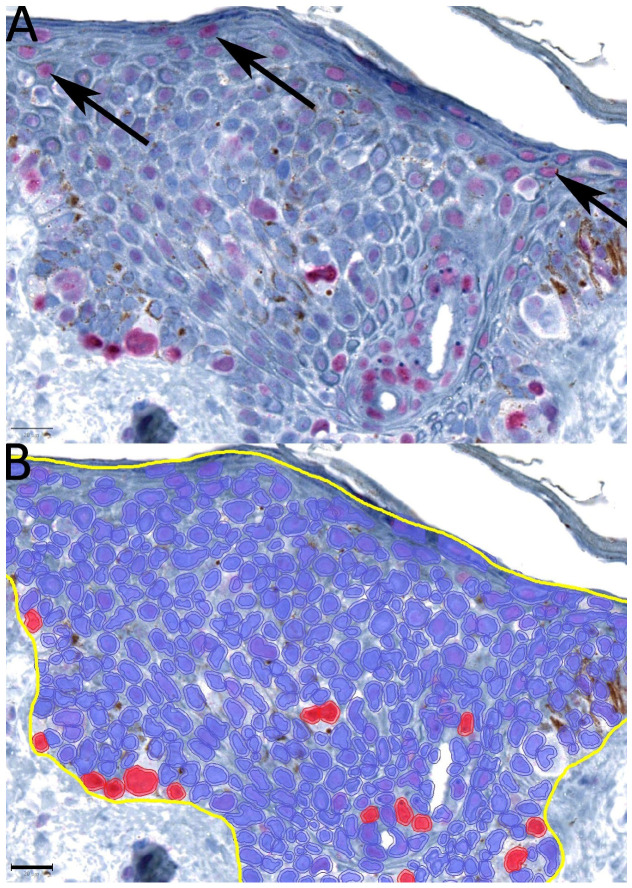
Definition of the staining threshold for each specimen. As the first two cell layers underneath the stratum corneum in the upper spinous layer of the epidermis usually show slight nuclear staining for 5-hmC (picture (**A**), black arrows), the threshold for positive-cell detection was set accordingly, i.e., only cells with a stronger density and expression of 5-hmC were considered as 5-hmC-positive cells (picture (**B**), the blue cells are considered to be 5-hmC-negative and the red cells to be 5-hmC positive). Black scale bar = 20 µm.

Overall, 105 slides were digitized. After an initial review of the selected cases, seven slides were excluded due to poor quality (five due to extensive bleaching and two due to other artifacts). Annotation and positive-cell detection were performed on 98 slides with QuPath (Figure 1). Three distinct annotations were performed for each slide for the epidermal with junctional component, dermal component, and entire lesion (Figure 2). Subsequently, the intensity threshold was set for each slide, taking into account slight positive staining of the keratinocytes several cell layers beneath the corneal layer (Figure 3). Overstaining was observed in six specimens, which was evident by the high reactivity of these epithelial cells. These slides were also excluded.

Finally, immunohistochemical stains for 5-hmC from a total of 92 different specimens were included in the analysis: overall, 42 (45.7%) SSM, including 11 (11/42) in situ melanomas and 50 (54.3%) DN. The mean tumor thickness of the invasive SSM was 0.42 mm (SD 0.2 mm). All DN were compound nevi. Additional clinical features are summarized in Table 1.

**Figure 4:**
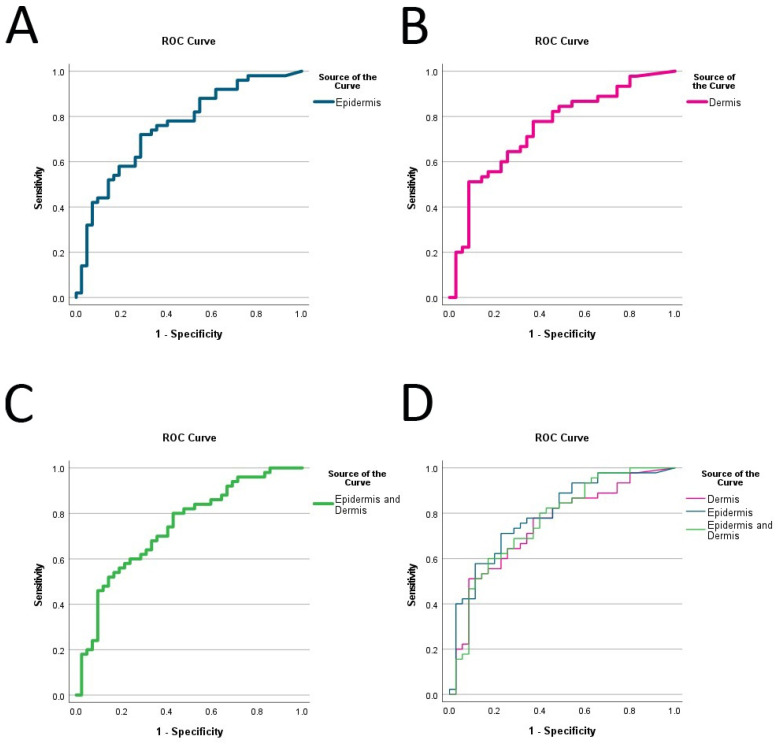
The area under the curve (ROCAUC) of the epidermis and dermo-epidermal junction (turquoise), the dermal component only (pink), and the entire lesion (epidermal, junctional, dermal, green). (**A**) ROCAUC of the epidermis (0.79, CI 95% 0.69–0.89); (**B**) ROCAUC of the dermis (0.74, CI 95% 0.63–0.85); (**C**) ROCAUC of the entire lesion (0.76, CI 95% 0.65–0.87); (**D**) ROCAUCs of picture (**A**–**C**) merged.

The receiver operating characteristic/area under the curve (ROCAUC) of 5-hmC-positive cells of the epidermis and dermo-epidermal junction was 0.79, for the dermis 0.74, and for the entire lesion 0.76 (Table 2, Figure 4).

The mean of 5-hmC-positive cells/mm^2^ for the epidermis and dermo-epidermal junction was 117.9 (SD 269.3) in SSM and 308.6 (SD 342.4) in DN and differed significantly (*p* = 0.002). In the dermal component, the mean was 459.7 (SD 1628.1) in SSM and 942.2 (SD 1253.9) in DN and showed a trend towards lower 5-hmC expression in melanomas (*p* = 0.069). In the entire lesion, the mean was 201.4 (SD 508.9) in SSM and 490 (SD 562.7) in DN (*p* = 0.006).

## 3. Discussion

The prognostic significance of 5-hmC was assessed for many different entities [15]. In patients with epithelial ovarian cancer, higher levels of 5-hmC were associated with a better prognosis, suggesting that 5-hmC could serve as a positive prognostic marker [11]. In gastric cancer and laryngeal squamous cell carcinoma, 5-hmC can also serve as an independent predictor of a poor prognosis [16,17]. Yang et al. suggested that epigenetic modifications influence gene expression and cellular processes, and these modifications have been associated with cancer development and progression. Patients with decreased levels of 5-hmC experienced poorer overall survival rates and had a higher likelihood of disease progression compared to patients with higher levels of 5-hmC [16]. Further, it was shown that loss of 5-hmC can be an additional valuable marker, alongside BAP1 and MTAP immunostains, for distinguishing diffuse malignant peritoneal mesothelioma from reactive mesothelial hyperplasia in peritoneal biopsies [18]. The combination of these markers could enhance the accuracy of diagnosis and aid in the differentiation between the two conditions [19]. Notably, the expression levels and distribution of TET enzymes and 5-hmC also showed higher expression in trophoblast villi from women with normal pregnancies compared to those who experienced early pregnancy loss. These findings suggest a potential link between the dysregulation of TET enzymes including 5-hmC and early pregnancy loss [20]. TET enzymes themselves are immediately involved in the process of DNA methylation. The expression of TET enzymes, particularly of the three members TET1, TET2, and TET3, can vary between melanocytes and melanoma in different stages. It is still unclear whether the loss of 5-hmC as a hallmark of melanoma represents a secondary association with disease progression or whether it directly contributes to malignant transformation. In a mouse model with NRAS-driven melanomagenesis, Tet2 cooperated with oncogenic NRASQ61K to promote melanoma initiation while suppressing specific gains in 5hmC. The authors concluded that TET2 acts as a barrier to melanoma initiation and progression, partly by promoting 5hmC gains in specific gene bodies [21]. DNA methylation-induced silencing of TET2 and TET3 resulted in an epithelial-to-mesenchymal-like process and promoted the metastasis of melanoma [22].

However, all mentioned findings show that these epigenetic modifications, and especially 5-hmC as an important intermediate in the DNA demethylation process, are crucial for gene expression and cellular processes, and these modifications are associated with cancer development, progression, and even pregnancy loss.

In distinguishing various melanocytic proliferations, 5-hmC has shown its diagnostic value, including benign nevi and melanomas. Melanocytic proliferations encompass a wide range of skin lesions that involve abnormal growth of melanocytes. Accurate diagnosis and differentiation of these lesions are crucial for appropriate clinical management and prognosis. In a study, a series of melanocytic proliferations was analyzed, including benign nevi and malignant melanomas, using 5-hmC immunohistochemistry to determine its diagnostic value. All examined blue nevi with epithelioid change (4/4), combined nevi (12/12), and deep penetrating nevi (5/5) had a strong 5-hmC nuclear reactivity. Meanwhile, 5/7 atypical deep penetrating nevi cases and 8/10 melanocytic tumors of uncertain malignant potential showed low or intermediate 5-hmC expression, and 8 heavily pigmented blue nevus-like melanomas as well as 7/8 pigmented epithelioid melanocytomas had no 5-hmC immunoreactivity [8]. The authors suggest that 5-hmC immunostaining patterns can distinguish between benign nevi and malignant melanocytic growth. The sensitivity and specificity of this assay for nevus vs. melanoma were 92.74% and 97.78%, respectively [8]. However, the number of tissue specimens examined was very small for drawing sufficient conclusions. Lian et al. examined 30 benign nevi and found a strong nuclear 5-hmC staining, whereas tumor cells in primary (*n* = 15) and metastatic (*n* = 10) melanomas showed partial or complete loss of 5-hmC. These findings were confirmed with a T4 phage β-glucosyltransferase-mediated 5-hmC glycosylation and an anti-5-hmC antibody-based dot-blot assay [12]. In another study, 12 proliferative nodules and 13 melanomas arising in giant congenital nevi were evaluated. It was found that 90.65% of the proliferative nodules had high 5-hmC expression levels, whereas the melanomas showed almost a complete loss (92.13%) [23]. Also, conjunctival melanomas (*n* = 37) displayed a significant reduction or absence of 5-hmC compared to normal conjunctival nevi (*n* = 40) (5-hmC expression: 54% vs. 100%, respectively) [24]. To achieve higher diagnostic precision, Siref et al. performed a dual 5-hmC/Melan-A immunohistochemical staining in 41 metastatic melanomas and 20 nodal nevi and found a total or partial loss of nuclear expression of 5-hmC in 97.56% of melanomas, while 100% of nodal nevi showed a strong expression [25]. Altogether, 5-hmC is increasingly lost in dysplastic melanocytic nevi and frequently absent in melanomas, which we confirm in our current findings. Thus, 5-hmC seems to be a suitable target to distinguish between benign and malignant melanocytic lesions of the skin.

However, most of these publications have in common that they focused on one marker and the comparison between healthy and malignant tissue. They did not include melanocytic dysplastic (considered still benign) proliferations. The distinction between dysplastic nevi and melanomas remains a major challenge faced by virtually all practicing dermatopathologists. Further, most published studies used a manual score for the interpretation of 5-hmC immunoreactivity, which is not standardized and may therefore bear some pitfalls. Thus, to minimize the intra- and inter-observer variability, we use a standardized computer-assisted assessment of 5-hmC staining to distinguish between SSM and DN.

Compared to the observation by Rodic et al., who compared the 5-hmC expression in nevi (not dysplastic) and melanoma with a manual score (ROCAUC = 0.784), the performance of our classification model is slightly improved [8]. However, the performance of ROCAUC < 0.8 (epidermal ROCAUC = 0.79, entire lesion ROCAUC = 0.76) lacks diagnostic accuracy compared to other markers such as PRAME. We evaluated PRAME in DN and SSM and presented ROCAUC values of >0.87 [6]. According to this data, we suggested that a high PRAME expression of more than 100 cells/mm^2^ is a strong indicator for melanoma, with a specificity of >87%, which is in comparison more reliable than 5-hmC [6]. These findings are in line with Rawson et al. [7], who investigated a manual score of PRAME, 5-hmC, and p53 expression in tissue microarrays of 53 benign nevi (including 27 dysplastic nevi) and 280 invasive cutaneous melanomas including a variety of subtypes. They also found a significant difference in 5-hmC expression but the sensitivity, specificity, and positive and negative predictive values were inferior to PRAME [7].

Notably, we found that the epidermal expression of 5-hmC is more valuable compared to dermal expression in differentiation between DN and SSM. To the best of our knowledge, this has not been previously described but should be kept in mind when interpreting the staining pattern of 5-hmC. According to our data, epidermal expression has a higher significance in the context of the classification of melanocytic lesions. This has also been observed for other immunohistochemical markers [6,26]. Thus, intra-epidermal melanocytes—considering subcorneal keratinocytes having a modest 5-hmC expression—should especially be carefully analyzed for 5-hmC expression to facilitate melanoma diagnosis in addition to morphologic features and potentially further immunohistochemical markers.

Limitations of this study were the fact that the blinding was impaired, as the investigator could recognize the diagnosis of the samples due to the overall morphology. Further, we evaluated exclusively borderline cases where immunohistochemical staining was performed retrospectively to facilitate melanoma diagnosis. This might have led to an impaired classification model. The SSM cohort included rather thin tumors that may introduce some bias, in particular regarding the evaluation of the epidermal and dermal components. The annotation of melanocytic cells was performed on morphologic grounds only and did not rely on a counterstain for a melanocytic antigen such as Melan A or SOX-10. This approach raises the possibility that some cells were not correctly annotated, particularly in the dermal components where melanocytic cells may be confused with immune infiltrate or endothelial cells.

## 4. Materials and Methods

This study was conducted under the approval of an independent research ethics committee of the Friedrich-Alexander-University Erlangen-Nuremberg (approval number 22-368-Br). This retrospective analysis was based on 92 cases in which immunohistochemistry was stained for the classification of SSM and DN between September 2022 and February 2023. The histological slides were obtained from the archives of the Unit for Dermatopathology, Department of Dermatology, Uniklinikum Erlangen, Germany. Each diagnosis was confirmed by a panel of at least two dermatopathologists. Lesions of unclear malignant potential were omitted and not included.

### 4.1. Immunohistochemistry

**Figure 5:**
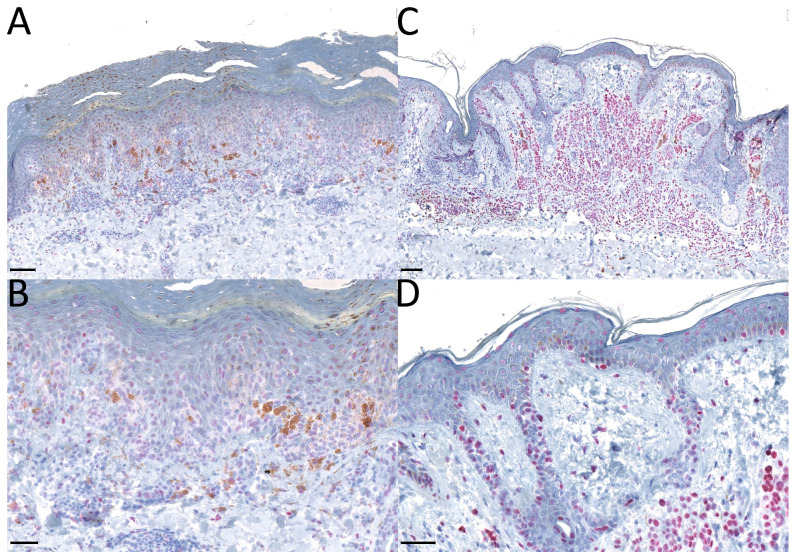
This figure shows an overview of immunohistochemically stained slides with 5-hmC antibody mounted with Fast Red chromogen. Picture (**A**,**B**) shows a superficial spreading melanoma, and picture (**C**,**D**) shows a dysplastic compound nevus, each with an overview ((**A**,**C**), black scale = 100 µm) and a higher magnification ((**B**,**D**), black scale = 50 µm).

IHC was performed in the certified laboratory of the Unit for Dermatopathology (Department of Dermatology, Uniklinikum Erlangen, Erlangen, Germany) using a fully automated IHC slide staining instrument (BenchMark XT by Roche Diagnostics, Rotkreuz, Switzerland). A commercially available antibody to 5-hmC (RRID: AB_10013602; Active Motif, Tokyo, Japan) with a dilution of 1:12.000 and an incubation time of 44 min (37 °C) was used. Further, Fast Red chromogen for immunohistochemical staining was applied to rule out interference with unspecific cytoplasmic melanin pigment (Figure 5).

### 4.2. Image Data Acquisition

All slides were digitized with the NanoZoomer S360 (Hamamatsu Photonics K. K.; Herrsching, Germany) at 40× magnification, with a resolution of 0.23 µm/pixel. The 5-hmC-stained slides were analyzed with the bioimage analysis software QuPath (version 0.3.2) [27]. The analysis was performed by one investigator (E.A.T.K.) who was blinded for the final histological diagnosis. Patients were sampled and information was obtained from the clinical and pathologic records. Prior to the first analysis (positive-cell detection), the digitized slides were reviewed for the sake of quality control to exclude bleached and artifact-rich specimens. In QuPath, annotations were performed of the epidermis (including the dermo-epidermal junction), as well as exclusively of the dermal component. For the detection of 5-hmC-positive cells, a threshold was established for the parameter of the nucleus, Fast Red optical density mean. Through QuPath, all cells in the annotated area were identified and distinguished between 5-hmC positives and negatives, according to the established threshold for each specimen. Precisely, from a specified density of Fast Red chromogen onwards, the cells were assessed as positive. Notably, as the first two cell layers beneath the stratum corneum are usually 5-hmC-positive, the threshold only recognizes those cells with a stronger intensity compared to this staining as an internal reference.

Prior to analysis, the slides were reviewed again to exclude specimens in which the set threshold recognized the subcorneal epithelial cells as positive (overexpression). Afterward, the score of positive cells/mm^2^ for all annotations was extracted.

### 4.3. Statistical Analysis

The evaluation cohort (*n* = 92) was split according to the diagnosis (SSM versus DN), and the mean number of positive cells/mm^2^ was compared using the t-test. A two-sided *p*-value of < 0.05 was considered statistically significant. Additionally, the evaluation cohort was classified through receiver operating characteristic curves and the area under the curve (ROCAUC). A ROCAUC score of >0.8 was considered a good and >0.9 a very good result [28]. All statistical analyses were performed using IBM^®^ SPSS Statistics (version 28.0.0.0, 190; Armonk, NY, USA).

## 5. Conclusions

In conclusion, 5-hmC immunostaining can be used as an ancillary tool to distinguish between DN and SSM; however, as a sole marker, 5-hmC may lack diagnostic reliability compared to other markers like PRAME. Our study highlights that assessing the epidermal with junctional staining intensity is slightly more accurate than assessing the dermal component. Lesions with a moderate 5-hmC expression and indifferent morphologic features will remain a challenge for dermatopathologists and warrant further immunohistochemical stainings such as PRAME or p16, which may facilitate a correct diagnosis, without mentioning other ancillary methods such as molecular pathology. Nevertheless, standardized evaluation of immunohistochemical stainings has great potential for the histologic assessment of melanocytic lesions.

## Figures and Tables

**Table 1:** Characteristics of the study cohorts.

Evaluation Cohort	Superficial Spreading Melanoma	Dysplastic Compound Nevus
N (%)	42 (45.7%)	50 (54.3%)
Vertical tumor thickness	Mean: 0.42 mm	
	Standard deviation: 0.2 mm	
Location	Head/Neck: 1 (2.4%)	Head/Neck: 5 (10%)
	Trunk: 21 (50%)	Trunk: 32 (64%)
	Upper extremity: 9 (21.4%)	Upper extremity: 7 (14%)
	Lower extremity: 11 (26.2%)	Lower extremity: 6 (12%)
Gender	Female: 20 (47.6%)	Female: 30 (60%)
	Male: 22 (52.4%)	Male: 20 (40%)
Age	Mean: 56 years	Mean: 44.34 years
	Standard deviation: 17.7	Standard deviation: 18.1

**Table 2:** Area under the curve.

Test Result Variable(s)	Area	Std. Error	Asymptotic Sig.	95% Confidence Interval	
				Lower Bound	Upper Bound
Epidermis/Junction	0.79	0.051	<0.001	0.69	0.89
Dermis	0.74	0.056	<0.001	0.63	0.85
Entire lesion	0.76	0.054	<0.001	0.65	0.87

## Data Availability

Data are contained within the article.
